# Frailty, MRI, and FDG-PET Measures in an Australian Memory Clinic Cohort

**DOI:** 10.3389/fmed.2020.578243

**Published:** 2021-01-14

**Authors:** Nan Jordan, Matthew Gvalda, Ross Cody, Olivia Galante, Cilla Haywood, Paul Yates

**Affiliations:** ^1^Department of Geriatric Medicine, Austin Health, Heidelberg, VIC, Australia; ^2^Department of Medicine, Eastern Health, Box Hill, VIC, Australia; ^3^Department of Medicine, University of Melbourne, Heidelberg, VIC, Australia; ^4^Neuropathology and Neurodegeneration Laboratory, Florey Neuroscience Institute, Heidelberg, VIC, Australia

**Keywords:** positron emission tomography (PET), dementia, magnetic resonance imaging (MRI), memory clinic, frailty, neurodegenerative disease, cerebrovascular disease, Alzheimer's disease

## Abstract

Given that the global population is aging, the number of age-related syndromes, such as frailty, is expected to rise in conjunction. Frailty is characterized by the loss of homeostatic reserve, rendering the individual vulnerable to poor health outcomes. Many biological mechanisms have been proposed to contribute to frailty. However, few studies have assessed the associations between frailty and brain diseases or neuroimaging biomarkers.

**Aims:** The aims of this study were to measure the prevalence of frailty in a memory clinic and to examine associations between frailty and brain changes found on magnetic resonance imaging (MRI) and 18-F deoxyglucose (FDG) positron emission tomography (PET) in memory clinic attendees.

**Methods:** A 54-items Frailty Index was retrospectively assessed for all clinic attendees from 2014. Frailty was defined as FI > 0.25. MR images were analyzed for stroke, cerebral small vessel disease [CSVD, including cerebral microbleeds (CMBs), cortical superficial siderosis (CSS), and white matter hyperintensity (WMH)], and neurodegenerative changes [MRI: mesial temporal atrophy (MTA), FDG-PET: regional hypometabolism], blind to clinical findings.

**Results:** There were 209 clinic attendees in 2014, of whom 121 had MRI performed. The prevalence of frailty (using FI) in the memory clinic in 2014 was 38.3% overall (patients without MRI: 43.2%, patients with MRI 34.7%, *p* = 0.25). Frailty was associated with presence of deep WMH, increased severity of periventricular WMH, and presence of CSS, but not neurodegeneration markers (MTA atrophy/FDG-PET hypometabolism).

**Conclusion:** The findings support the idea that previously reported associations between frailty and imaging evidence of CSVD in other cohorts are also relevant to the Australian clinic setting. Given that a large proportion of memory clinic attendees are frail, there may be opportunities for interventions to reduce preventable adverse health outcomes, such as falls and fractures, and reduce the prevalence and impact of frailty in this cohort.

## Introduction

Frailty is increasingly recognized as an important contributor of morbidity and mortality in the geriatric population (1). It is characterized by the loss of homeostatic reserve, rendering the individual vulnerable to minor stressors and subsequent adverse health outcomes including death (2). It is estimated that one in 10 individuals aged >65 years old in the community setting are frail (3). In addition, some contributors to frailty and frailty-associated harm may be reversible [e.g., sarcopenia, malnutrition, social isolation, osteoporosis, polypharmacy, and others (4)]. Therefore, predicting frailty and ways to mitigate its risks is an important area in geriatric medicine.

The most common ways to measure frailty are as a physical phenotype (e.g., Fried's frailty phenotype) (5) or multidimensional scales such as a Frailty Index (FI) (6) or the Edmonton Frailty Scale (EFS) (7). “Cumulative-deficit” FIs are derived by averaging a specified number of health deficits, to describe frailty as a continuous index from zero (no deficits) to one (maximum number of deficits). Rockwood et al. recommend that health variables chosen should accumulate with age, not saturate too early, be associated with adverse health outcomes, and cover a range of systems (6, 8). Including 30–40 items maintains power and accuracy of the scale (8). The EFS encompasses nine domains and categorizes individuals as robust, vulnerable, and frail (7). Both the FI and EFS recognize that frailty may be the result of the malfunction of multiple health-related systems (7, 9). Critical to these scales is the cumulative burden, rather than the type, of systems involved (2).

There is mounting evidence supporting an association between frailty and the loss of reserve in the musculoskeletal, endocrine, immune, and cardiovascular systems (10). It has also been suggested that an underlying mechanism driving these associations may be chronic inflammation, as evidenced by increased TNF-alpha, IL-6, IFN-gamma, and C-reactive protein levels in frail patients (4, 11). Chronic inflammation has also been linked to frailty via disruption of several systems that affect the central nervous system such as the endocrine and immune system (12).

Diseases associated with accumulation of brain pathology may also be associated with dysregulation of multiple systems including chronic inflammation, as well as result in loss of physical function, cognitive changes, and increased susceptibility to illness. Brain pathology can be broadly divided into cerebrovascular disease and neurodegenerative changes. Cerebral small vessel disease (CSVD) refers to neuroimaging and pathological findings associated with the consequences of dysfunction of perforating arterioles, venules, and capillaries of the brain (13). Imaging evidence of CSVD includes deep brain or “lacunar” infarcts, white matter hyperintensities (WMHs), and cerebral microbleeds (CMBs) (14).

Neurodegeneration is characterized by sequential malfunction and loss of neurons (15). The most common neurodegenerative disorder is Alzheimer's disease (AD), also the most prevalent type of dementia (16). Atrophy in the mesial temporal lobe (hippocampal region) is recognized to be associated with memory loss and dementia due to AD (17). In addition, positron emission tomography (PET) and single-photon emission computed tomography (SPECT) can demonstrate abnormal neuronal function (via blood flow and oxygen or glucose uptake) in AD and other neurodegenerative diseases (16).

Frailty has also been linked to cognitive impairment and dementia (18, 19). People with dementia experience physical as well as cognitive symptoms, with weight loss, gait disorder, and falls, as part of the clinical course of the disease (20, 21). Presence of AD brain biomarkers have been associated with falls and weight loss, even before cognitive symptoms emerge (21). So, it follows that people with neurodegeneration or cerebrovascular disease on brain imaging may have increased prevalence of frailty. A number of studies have assessed the associations between frailty and cerebral changes using different neuroimaging modalities (22–37). However, due to differences in their study design, frailty measures employed, imaging modalities, and population studied, direct comparison should be carried out with caution. This study aims to determine prevalence of frailty in Australian memory clinic patients and whether imaging evidence of cerebrovascular disease or neurodegeneration are associated with frailty in this setting.

## Materials and Methods

### Study Design

This is a cross-sectional retrospective file audit of patients presenting to the memory clinic (2014), including clinical and imaging information. A frailty assessment (EFS) was also collected in a small prospective sample in 2018.

### Setting

Our Cognitive Dementia and Memory Service (CDAMS) is a large tertiary referral memory clinic, offering diagnostic services, multidisciplinary support, and education for people experiencing cognitive impairment.

Patients are typically referred to the clinic by their primary care physician; however, patients can also refer themselves if concerned about their cognition. Patients are then triaged by the CDAMS staff. As this is primarily a diagnostic service, those who already have a previously established diagnosis of dementia are referred elsewhere.

The multidisciplinary CDAMS team includes geriatricians, neurologists, psychiatrists, neuropsychologists, social workers, nurses, occupational therapists, and speech pathologists. Routine clinical assessment includes medical history as well as relevant physical, neurological and cognitive examinations. Patients typically undergo brain imaging, including MRI, computerized tomography (CT) brain, SPECT, and PET scans as part of the investigation workup. Finally, a multidisciplinary case conference is held where a consensus diagnosis is reached.

### Participants

All memory clinic attendees from 2014 (*n* = 209) were included in the retrospective audit cohort; 121 had MRI available for assessment. Those without MRI or with scans performed elsewhere were not included in analysis of imaging biomarkers.

Fifty-three patients between February 2018 and June 2018 underwent EFS measures during clinic attendance. There were no patients included under both cohorts.

### Data Collection

#### Patient Demographics

General information, including age, sex, education level, smoking and alcohol status, and consensus diagnosis at the memory clinic, was collected from electronic health records.

### Frailty Measurement

#### Frailty Index

A cumulative deficits FI was derived from a retrospective review of electronic records of clinic attendance for all patients. A total of 54 health variables (Appendix 1) were included based on previously published indices and information available. Health variables in this study included comorbidities, medication use, social support, functional status, recent falls, disability, and physical parameters (blood pressure, weight loss, etc.) (Appendix 1). Information regarding the health deficits was obtained from electronic health records, which included referral letter, medical summaries, and assessments made by members of the allied health team. A FI was calculated for each individual as a ratio between the number of health deficits present and the total number of health deficits measured, and frailty was defined as FI > 0.25, as previously published (6, 18).

#### Edmonton Frailty Scale

To determine robustness of the FI generated from retrospective file review, prospective frailty assessments using EFS were also collected in a convenience sample of patients from 2018. The EFS includes nine domains: cognition, general health status, functional dependence, social support, medication use, nutrition, mood, continence, and gait assessment (7). Frailty is scored out of 17, with a cutoff score of 6 or above used to stratify robust from vulnerable or frail individuals (7). The scale was administered as a questionnaire, which included two activities: drawing a clock and assessing timed-up and go (7). Patients were usually assessed in the company of their caregiver, who corroborated the patient's answers.

### Assessment of Brain Changes

#### MRI Acquisition

All patients with MRI (121 of 209) included in the analysis were scanned on a single 1.5-T Siemens Avanto scanner (Siemens, Erlangen, Germany), producing slices of 2.5 mm thickness (flip angle, 15°; echo time, 40 ms; and repetition time, 27 ms) at our hospital radiology department. Imaging acquired included the axial susceptibility-weighted image (SWI), 3D T1/MPRAGE, coronal T1 MRI, and axial T2/FLAIR (fluid-attenuated inversion recovery) following a standard protocol.

#### MRI Interpretation

MR images were read for the presence of CMBs and CSS by two trained readers, blind to clinical information. CMBs were identified as well-demarcated hypointense lesions, measuring 2–10 mm on T2-weighted MRI through the Microbleed Anatomical Rating Scale (MARS) (38). CSS was identified on SWI MRI as hypointense curvilinear lines located adjacent to the cortex of the brain (39). Results of both readers were compared, and a third reader with expertise in neuroimaging reviewed to clarify any disagreement between the first two readers. Inter-rater agreement was measured between raters.

Deep and periventricular WMHs were classified by a single experienced reader using the Fazekas scale, according to size, location, and confluence (40).

Atrophy in the mesial temporal lobe was assessed using the mesial temporal atrophy (MTA) scale. Atrophy was graded from 0 to 4, on coronal T1-weighted MRI by consensus of two readers, blind to clinical data. An abnormal MTA was interpreted as scores ≥2 in those <75 years old and scores ≥3 in those ≥75 years old (17). An intra- and inter-reader agreement was performed for reader 1 (*n* = 30) and between readers 1 and 2 (*n* = 30).

Presence of stroke was obtained from clinical radiologists' reports. Clinical reports of PET or SPECT imaging by molecular imaging physicians were categorized as normal, consistent with AD, frontotemporal lobar degeneration (FTLD), dementia with Lewy body (DLB), or other causes (e.g., appearances suggestive of stroke, subdural hematoma, mass lesion, and previous trauma).

### Statistical Methods

Statistical analysis was performed using the Statistical Package for the Social Sciences (SPSS, version 23.0). For continuous variables, normality assessment was performed using Shapiro–Wilk test and group comparisons were performed using Mann–Whitney *U*-test (non-parametric) or *t*-test (normal distribution). χ^2^ (Chi square) test was employed to compare categorical variables and determine associations between brain imaging findings and frailty. Kappa statistic (κ) was used to assess level of agreement between frailty assessed using retrospective FI and EFS and agreement between MRI readers for CMB and MTA. Data reported in the *Results* section refer to the 2014 clinic cohort unless specified.

### Ethics

The study was approved by our institution's Research Ethics Committee.

## Results

### Demographics

Demographics of patients are outlined in Table 1. The median age of participants was 76.0 years overall [interquartile range (IQR) 68–83] and there were more female than male patients overall (54.1 vs. 45.9%). Reasons for lack of MRI included presence of a pacemaker, patient deemed too frail, clinical diagnosis was felt sufficient, patient declined, and loss to follow-up. There was no significant difference in age, gender, education, smoking, or alcohol history between participants with and without MRI. People with MRI were more likely to have a diagnosis of MCI and less likely to receive “other” (including psychiatric) diagnoses (*p* < 0.01). People with MRI had lower frailty measures compared with those that did not undergo MRI [median FI = 0.30 (IQR 0.13–0.29) vs. 0.23 (0.16–0.30), *p* = 0.04].

**Table 1:** Demographic characteristics according to MRI status.

**Cohort**	**All patients**	**Without MRI**	**With MRI**	***p***
*N*	209	88	121	
Age, median [interquartile range (IQR)]	76.0 (68.0–83.0)	77.5 (70.0–83.5)	76.0 (66.0–83.0)	0.14
Women, *n* (%)	113 (54.1)	47 (53.4)	66 (54.5)	0.68
Frailty Index, median (IQR)	0.21 (0.14–0.30)	0.23 (0.16–0.30)	0.20 (0.13–0.29)	0.04
Frail, *n* (%)	80 (38.3)	38 (43.2)	42 (34.7)	0.21
Diagnosis, *n* (%)				<0.01
• Dementia (all-cause) • Mild Cognitive Impairment (MCI) • Subjective Cognitive Impairment (SCI) • Other	72 (34.4) 32 (15.3) 61 (29.2) 44 (21.1)	29 (33.0) 2 (2.3) 23 (26.1) 34 (38.6)	43 (35.5) 30 (24.8) 38 (31.4) 10 (8.3)	
Education, *n* (%)^*^				0.81
• None • Primary • Secondary • Tertiary	2 (1.3) 29 (18.7) 84 (54.2) 40 (25.8)	1 (1.6) 13 (20.3) 36 (56.2) 14 (21.9)	1 (0.8) 16 (13.2) 48 (39.7) 26 (21.5)	
Smoking, *n* (%)				0.84
• Never smoked • Former smoker • Current smoker	148 (70.8) 53 (25.4) 8 (3.8)	63 (71.6) 21 (23.9) 4 (4.5)	85 (70.2) 32 (26.4) 4 (3.3)	
Alcohol, *n* (%)				0.30
• Non-drinker • Previous heavy or current moderate drinker • Heavy (>4 standard drinks, most days)	177 (84.7) 23 (11.0) 9 (4.3)	72 (81.8) 13 (14.8) 3 (3.4)	105 (86.8) 10 (8.3) 6 (5.0)	
Interpreter used, *n* (%)	37 (17.7)	17 (19.3)	20 (16.5)	0.37

* *Missing data for education status, patients without MRI: 24, patients with MRI: 30*.

### Frailty Prevalence

The prevalence of frailty was 38.3% overall. Those with MRI had (non-significant) lower prevalence of frailty than those without (34.7 vs. 43.2%, *p* = 0.21).

Prevalence of frailty was similar in women (44/113, 38.9%) and men (36/96, 37.9%) (*p* = 0.89).

### Frailty Scale Agreement (2018 Patient Cohort)

There was significant, albeit modest agreement, between frailty assessed using FI and prospective EFS measures (κ = 0.54, *p* < 0.001, *n* = 53).

### Neuroimaging Biomarker Assessment

Of 121 patients with MRI, 6 did not have coronal views available for MTA rating. PET/SPECT scans were available for 88 clinic attendees only.

Of 121 patients with MRI, 27.3% (*n* = 33) presented with CMBs in the lobar, deep, or infratentorial regions; 82.6% (*n* = 39) had periventricular WMH and 76.9% (*n* = 93) had deep WMH of mild, moderate, or severe grade; and 2.5% (*n* = 3) presented with CSS. Seven (5.8%) had evidence of ischemic stroke. MTA was identified in 26.1% (30/115). Neurodegenerative findings (characteristic of AD, FTLD, or DLB) were present in 61.4% (54/88) with PET/SPECT scans conducted.

#### MRI Inter and Intra-Reader Agreement

##### Microbleed Rating

There was good inter-rater agreement between reader 1 and reader 2 (κ = 0.75, *p* < 0.001), and each had a very good agreement compared with reader 3 (κ = 0.84 for reader 1 and κ = 0.89 for reader 2, *p* < 0.001).

##### MTA Score Evaluation

There was good inter-rater agreement between reader 1 and reader 2 (κ = 0.75, *p* < 0.001) and very good intra-reader assessment for reader 1 (κ = 0.8, *p* < 0.001).

### Association Between Brain Findings and Frailty

Frailty (defined as FI > 0.25) was significantly associated with the presence of deep WMH of all types (mild, moderate, and severe) (*p* = 0.049), severe periventricular WMH (*p* < 0.05), and CSS (*p* = 0.023) (Table 2). There was also significant linear-by-linear association between frailty and periventricular WMH severity (*p* = 0.007), with prevalence of frailty increasing with increasing severity of periventricular WMH. Frailty was also associated with CSS (*p* = 0.023). The association between frailty and stroke approached but did not reach significance (*p* = 0.056).

**Table 2:** Associations between brain changes (found on MRI and SPECT/PET) and frailty status.

**Brain variables**	***N***	**Frailty status**		
		**Not frail, *n* (%)**	**Frail, *n* (%)**	***p*-value**
Stroke	120^a^	2 (2.7)	5 (11.1)	0.056
Cerebral microbleeds	121	18 (23.7)	15 (33.3)	0.249
• Lobar • Deep • Infratentorial		14 (18.4) 4 (5.3) 3 (3.9)	12 (26.7) 4 (8.9) 5 (11.1)	0.286 0.438 0.125
White matter hyperintensity (WMH)	121			
• Periventricular WMH^d^				0.051
◦ None ◦ Mild ◦ Moderate ◦ Severe		16 (21.1) 28 (36.8) 21 (27.6) 11 (14.5)	5 (11.1) 11 (24.4) 14 (31.1) 15 (33.3)^*^	
• PV WMH >1		60 (78.9)	40 (88.9)	0.16
• Deep WMH^d^				0.229
◦ None ◦ Mild ◦ Moderate ◦ Severe		22 (28.9) 38 (50.0) 14 (18.4) 2 (2.6)	6 (13.3)^*^ 25 (55.6) 12 (26.7) 2 (4.4)	
• Deep WMH >1		54 (71.0)	39 (86.7)	0.049
Cortical Superficial Siderosis	121	0 (0.0)	3 (6.7)	0.023
Mesial temporal lobe atrophy	115^b^	20 (28.2)	10 (22.7)	0.518
PET/SPECT diagnosis	88^c^			0.699
• Normal or non-specific • Neurodegenerative		21 (35.6) 38 (64.4)	13 (44.8) 16 (55.2)	

* *p < 0.05 using z-test for difference in proportions*.

a *One patient excluded due to large subarachnoid cyst causing temporal lobe mass effect*.

b *MRI in the coronal plane was only available for 115 patients*.

c *PET/SPECT scans were available for only 88 patients*.

d *Linear-by-linear association between FI and (p = 0.007) periventricular WMH severity and (p = 0.06) deep WMH severity*.

There was no association between frailty and CMBs (*p* = 0.249), MTA (*p* = 0.518), or PET/SPECT findings (*p* = 0.699).

## Discussion

This study aimed to measure frailty in an Australian memory clinic cohort and to describe the associations between frailty and brain changes found on MRI. We identified that 38.3% of all clinic attendees were frail. Frailty was associated with the presence of deep WMH of all types (mild, moderate, and severe), severe periventricular WMH, and CSS. There was a moderate agreement between frailty assessed prospectively (EFS) and by FI derived from medical record review.

### Frailty Measurement and Prevalence

The prevalence of frailty in this study was measured using both the FI and the EFS. Both are known as multidimensional scales. Some previous studies suggest that these types of frailty scales are better able to capture frailty holistically since it measures deficiencies across multiple systems (1), as opposed to a frailty phenotype (3). However, a disadvantage is that many studies differ in the health variables assessed and, hence, health systems measured, which may limit comparability between studies. In addition, it is difficult to compare prevalence in this study to those from previous studies reporting frailty and brain changes due to heterogeneity between populations and frailty measures. Moreover, some studies did not report their frailty prevalence (25, 27, 29, 30).

### Associations Between Brain Changes and Frailty

#### Cerebrovascular Changes and Frailty

Several studies have previously studied associations between frailty indices and markers of CSVD and atrophy (22–30). However, to the author's knowledge, this is the first study to also examine the associations between frailty and CSS. In this study, CSVD was associated with frailty, which is consistent with previous publications (23–31). In particular, associations were found between CSS, severe periventricular WMH, and presence of deep WMH.

A number of previous studies have demonstrated an association between frailty and WMH (24, 26–31, 33). Adding to this, a higher burden of WMH has also been linked to progression of frailty (27, 34). This may suggest that WMH could not only be used as a marker of frailty or its progression but also be viewed as an opportunity for therapeutic intervention such as targeting cardiovascular risk factors (27). However, more studies are required to further understand if targeting WMH could lead to a slower progression or reduced incidence of frailty.

Two studies with younger cohorts (>50 years old) did not find this association (22, 23). WMHs are common with increasing age: one study estimates that 90% of elderly individuals (>60 years old) exhibit WMH, often with a subclinical presentation (41). It is therefore expected that younger cohorts are not only less likely to be frail but also less likely to have WMH or demonstrate an association between them. Use of different frailty assessment may also explain differences in associations found. These two studies (22, 23) used the Fried's frailty phenotype (FFP). The FFP consists of five physical parameters, including weight loss, exhaustion, decreased physical activity, slow gait, and weakness (5). Measuring frailty by focusing on one system (musculoskeletal) may have led to less granularity and possibly to an underestimation of frailty in their cohorts. Therefore, this may have influenced the association between frailty and WMH.

The relationship between frailty and CSVD appears to be complex and possibly bidirectional. CSVD, particularly WMH, is associated with slowed gait, falls, disability, and other geriatric syndromes (41). Severe WMH doubles the risk of progression from a state of autonomy to becoming dependent, over the course of 3 years (42). WMH has also been linked to depression, cognitive decline, and urinary issues (42). In turn, geriatric syndromes are closely associated with frailty (43). Slowed gait in particular may lead to loss of independence, functional decline, and poor quality of life (44). In addition, decrease in physical activity has been associated with worsening WMH and cerebral atrophy (45). Furthermore, increasing physical activity has been shown to improve depressive symptoms, including fatigue (44).

Moreover, another two studies reported on the association between frailty and strokes (23, 26), while only one of them found frailty to be linked to CMBs (23). A third study reported no association between either CMBs or strokes and frailty (27).

This study also found an association between frailty and CSS, which may be due to cerebral amyloid angiopathy (CAA), hypertensive arteriopathy, trauma such as falls, and others (46). However, the number of CSS was small, and therefore, this finding should be interpreted with caution.

#### Neurodegenerative Changes and Frailty

There are several reports linking brain atrophy and frailty (22, 24, 25, 32), including both cortical and subcortical, global gray matter, and regional volume (hippocampal volume and other regions). We did not find an association between neurodegenerative changes, including MTA, and frailty. Given that mesial temporal lobe atrophy is associated with AD, the most common cause of dementia (16), and previous studies have linked dementia to frailty (47), one might have expected that MTA or neurodegenerative patterns on PET/SPECT imaging may have been associated with frailty in our study. Our findings are similar to Cheong et al., who reported no association between either global or MTA and frailty in those with early AD (finding instead an association with deep WMH) (35).

More accurate measurement (e.g., using volumetric MRI to determine hippocampal volume) rather than a visual rating scale would be helpful to further assess this. Unfortunately, the clinical imaging performed did not provide for three-dimensional volumetric analysis. Undertaking additional MTA ratings in an increased number of patients would also be beneficial to further evaluate these associations.

The underlying pathophysiology linking cerebrovascular and neurodegenerative changes, frailty, and cognitive decline is not well-understood. However, studies suggest that arterial stiffness may play a role in connecting frailty, WMH, and cognitive impairment (48). Arterial stiffness consists of microvessel arteriosclerosis in conjunction with vascular endothelial dysfunction. These abnormalities may be due to hemodynamic stress (blood–brain barrier breakdown or progression of arteriosclerosis) (49). In addition, insulin resistance has been linked to muscle atrophy, sarcopenia, and incidence of frailty, as well as incident AD (50).

### Limitations and Strengths

Our study was limited by a small sample size and its cross-sectional, retrospective design that limits inference of directionality of associations. Additionally, the highly selected memory clinic setting means that the findings of this study cannot be generalized to a wider population.

Both CSVD and frailty are known to be more prominent with increasing age. However, due to a small sample size, this study did not adjust for age among other possible confounders. Additional data collection in the future will permit multivariate analysis to further shed light on these associations.

Of the entire patient sample, 88 did not have MRI available. Excluding patients without MRI may have meant that those with greater frailty and possibly with a higher burden of CSVD and neurodegenerative changes were not included in the study. Hence, this may have led to underestimation of prevalence and may have affected the association between brain changes and frailty. To support this, those without MRI had slightly—but significantly—higher frailty indices (although the prevalence of frailty did not differ between the two groups).

Construction of the FI was limited to the data available on retrospective audit of the electronic health record, vulnerable to inconsistencies in documentation and missing data (assumed to be absent health deficits), which could bias the findings. The EFS, although measured prospectively, relied on patient insight and truthfulness, although attempts were made to verify responses with their caregivers in order to capture a more accurate picture of their health status. The EFS also specifically includes assessment of cognitive performance and is more greatly influenced by cognitive changes, whereas the authors selected FI items to minimize cognition-dependent health variables, which may explain the modest magnitude of correlation observed between the two scales in this study.

## Conclusion

This study combined comprehensive multimodal neuroimaging to examine the associations between brain changes and frailty. To the author's knowledge, this is the first study to assess functional (PET and SPECT imaging) evaluation of neurodegeneration and include CMB and CSS with other markers of CSVD in analyses of associations with frailty. The findings of this study build on previous publications showing an association between frailty and imaging evidence of CSVD, in particular WMH. This study also found an association between frailty and CSS, which may be due to angiopathy or trauma from falls. Larger prospective longitudinal studies are warranted in order to better appreciate any causal relationships and account for important confounders. Given that a large proportion of memory clinic attendees are frail, there may be opportunities for interventions such as targeting vascular risk factors, promoting physical activity, and other lifestyle or diet interventions to potentially reduce the impact of frailty, or proactive treatment of osteosarcopenia in this cohort to reduce falls and fracture risk.

## Data Availability Statement

The raw data supporting the conclusions of this article will be made available by the authors, without undue reservation.

## Ethics Statement

The studies involving human participants were reviewed and approved by Austin Health Human Research Ethics Committee. Written informed consent for participation was not required for this study in accordance with the national legislation and the institutional requirements.

## Author Contributions

PY, CH and NJ were responsible for the planning, design of the study, and statistical analysis. NJ and MG were involved in data collection, data analysis, and wrote sections of the manuscript. NJ, PY, RC, and OG performed imaging reading. All authors contributed to manuscript revision, read, and approved the submitted version.

## Conflict of Interest

The authors declare that the research was conducted in the absence of any commercial or financial relationships that could be construed as a potential conflict of interest.
