# Supplementary material for: Frailty, MRI, and FDG-PET Measures in an Australian Memory Clinic Cohort
Source: Front Med (Lausanne). 2021 Jan 14;7:578243. doi: 10.3389/fmed.2020.578243 (PMC7840574; doi:10.3389/fmed.2020.578243)
Supplement: Supplementary file 1 [file Table_1.docx]

**Appendix 1.** Frailty Index (54 health deficits)

| Help dressing | Eyesight issues | Skin problems |
| --- | --- | --- |
| Help bathing | Hearing issues | Other MSK |
| Help grooming | Dental problems | Other medical issues |
| Help toileting | Eye problem | Lives alone |
| Help eating | Ear problem | Weight loss >4.5kg in the last year |
| Help using phone or remote control | Head and neck issues | Impaired mobility/mobility aids |
| Help with housework | Chest problems | Systolic BP <90 or >140 |
| Help with finances | Chronic lung disease | Diastolic BP <60 or >90 |
| Help with meal preparation | Respiratory issues | Sleeping issues |
| Help taking medications | GIT problem | Thyroid issues |
| Help with shopping | Constipation | Hypertension |
| Help to get to places out of walking distances | Abdominal issue | Hyperlipidaemia |
| Medications >5 | Malignancy | Myocardial Infarction |
| >1 Falls in the last year | Osteoporosis | Congestive Heart Failure |
| Urinary incontinence | Arthritis | Arrhythmia |
| Bowel incontinence | Spinal issues | Vascular issues |
| Rectal problems | Trouble with feet or ankles | Chronic renal disease |
| Genito-urinary problems | Trouble with nerves | Diabetes Mellitus |
